# Supplementary material for: Cryogenic characteristics of graphene composites—evolution from thermal conductors to thermal insulators
Source: Nat Commun. 2023 Jun 2;14:3190. doi: 10.1038/s41467-023-38508-3 (PMC10238409; doi:10.1038/s41467-023-38508-3)
Supplement: Supplementary file 1 — Supplementary Information [file 41467_2023_38508_MOESM1_ESM.pdf]

Supporting Information

Cryogenic Characteristics of Graphene Composites – Evolution from Thermal Conductors to Thermal Insulators

Zahra Ebrahim Nataj,[1] Youming Xu,[2] Dylan Wright,[1] Jonas O. Brown,[1] Jivtesh Garg,[3] Xi Chen, [2] Fariborz Kargar,[1,*] and Alexander A. Balandin[1,*]

[1]Nano-Device Laboratory and Phonon Optimized Engineered Materials Center, Department of Electrical and Computer Engineering, University of California, Riverside, California 92521 USA

[2]Department of Electrical and Computer Engineering, University of California, Riverside, California 92521 USA

[3] Department of Aerospace and Mechanical Engineering, University of Oklahoma, Norman, Oklahoma 73019 USA

[*] Corresponding authors: fkargar@ece.ucr.edu ; balandin@ece.ucr.edu ; web-site: http://balandingroup.ucr.edu/

Contents

1 Sample preparation 2
2 Mass density measurements 3
3 Scanning electron microscopy characterization of overlapping fillers 4
4 Heat capacity measurements and uncertainty analysis 4
5 Thermal conductivity measurements and uncertainty analysis 6
6 Durability of composites at cryogenic temperatures 8
7 Effective medium model for cryogenic heat conduction in low-loading composites 9
8 Effective medium model for cryogenic heat conduction in high-loading composites 12

1 Sample preparation

Several composite samples were prepared by mixing precalculated quantities of the epoxy resin, bisphenol A (epichlorohydrin) with a molecular weight of 700 (Allied HighTech Products, Inc., the USA), the hardener, triethylenetetramine (Allied HighTech Products, Inc., the USA), and few-layer graphene (FLG) fillers (xGnP H-25, XG Sciences, the USA) to hit a targeted filler loading level. The average lateral dimension and surface area of the FLG fillers were 25 μm and 65 m^2g^{-1}, respectively. In order to have a uniform compound, FLG was added in several steps and mixed for 3 minutes at 800 rpm in a high-shear speed mixer (Flacktek, Inc., the USA). The hardener was then added to the epoxy resin at a mass ratio of 12:100. The final compound was mixed and vacuumed for 10 minutes to remove any possible trapped air bubbles. The latter was performed three times to achieve void-free composites. The samples were then poured into silicon molds and left at room temperature for about 8 hours to cure and solidify. At higher graphene concentrations, the samples were slightly pressed. Finally, all samples were heated at 130℃ in a furnace for 3 hours. The final composite samples were disks with a diameter of 25.4 mm and a thickness of 5 mm. The optical images of the samples are shown in Supplementary Figure 1.

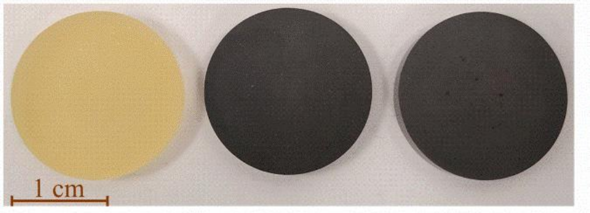

Supplementary Figure 1. Sample characterization. From left to right, optical images of pristine epoxy and composites with 2.6 vol% and 5.4 vol% loading of few-layer graphene.

2 Mass density measurements

The mass density of the samples was determined using the Archimedes principle and an electronic scale (Mettler-Toledo LLC, the USA). The density is calculated using the equation below:

$$\rho_c = (w_a/(w_a - w_w)) \times (\rho_w - \rho_a) + \rho_a \tag{S1}$$

where w_a, w_w are the sample's weight in air and water, respectively, and ρ_w and ρ_a are the density of deionized water and air at room temperature. The results of the mass density measurements for different filler concentrations are presented in Supplementary Figure 2.

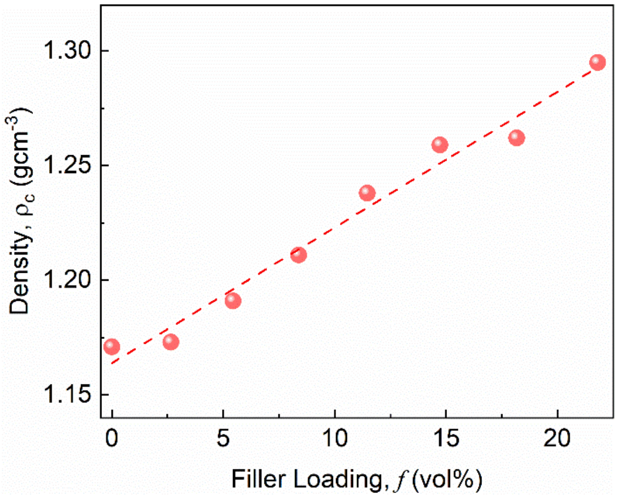

Supplementary Figure 2. Mass density of composites as a function graphene filler loading. The mass density of composites changes linearly as a function of filler loading confirming negligible porosity of the prepared composites.

3 Scanning electron microscopy characterization of overlapping fillers

As the volume fraction of fillers increases beyond a certain loading, referred to as the percolation threshold, the fillers start to overlap. At and beyond the percolation regime, fillers create a network of electrically and thermally conductive pathways within the base polymer matrix. Supplementary Figure 3 shows an SEM image of overlapping fillers in the composite with 21.7 vol% graphene loading.

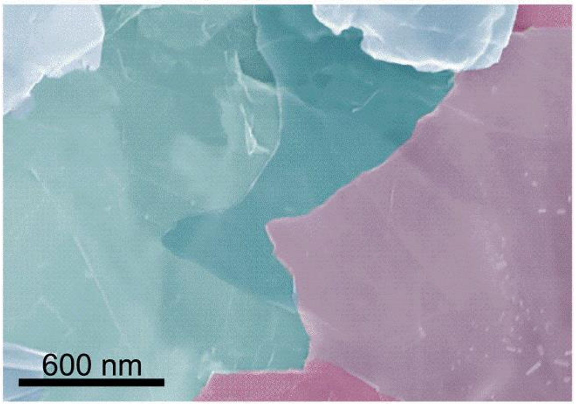

Supplementary Figure 3. Percolation in composite samples. SEM image of an epoxy sample with 21.7 vol% graphene loading. Note the regions where the fillers overlap.

4 Heat capacity measurements and uncertainty analysis

For the heat capacity measurement with the PPMS method, 5 mg of each composite sample was used. The measurements were conducted in the temperature range of 2 K to 300 K. In all measurements, one should consider that the temperature of the sample, T_s, is different from the temperature of the platform, T_p, due to the unavoidable thermal contact between the adjoining surfaces.[1] The heat transfer between the sample and the platform is described by the following equations:

$$C_{platform}\frac{dT_p}{dx} = P(t) - K_w\left(T_p(t) - T_b\right) + K_g(T_s(t) - T_p(t)) \quad \text{(S2)}$$

$$C_{sample}\frac{dT_s}{dx} = -K_g(T_s(t) - T_p(t)) \quad \text{(S3)}$$

where $P(t)$ is the heater power and $C_{platform}$ and C_{sample} are the heat capacity of the platform and the sample, respectively. K_w and K_g are the thermal conductance of the supporting wires and the thermal conductance between the platform and the sample in the presence of the grease layer. T_b is the temperature of the thermal bath. Note that T_s and T_p are functions of time. The error of the heat capacity measurements includes the total heat capacity error from the fitting of the heat capacity parameters, the addenda heat capacity error from the addenda measurement, the sample mass error, and the fitting deviation error from the modeling. Therefore, the error in heat capacity C_p is calculated as

$$\sigma(C_p) = C_p \times \sqrt{(\frac{R_{C_{total}}}{C_{total}})^2 + (\frac{R_{C_{platform}}}{C_{platform}})^2 + (\frac{\Delta M}{M})^2 + (\frac{R_{\Delta T}}{\Delta T})^2} \quad \text{(S4)}$$

where $R_{C_{total}}$ and $R_{C_{platform}}$ are the mean-square deviation of the fit to variations in the total heat capacity and platform heat capacity, M is the mass of the sample and $R_{\Delta T}$ is the fitting deviation error. Supplementary Figure 4 shows the heat capacity of all samples as a function of temperature in the range of $100\ \text{K} \leq T \leq 300\ \text{K}$. One can see that the heat capacity of each sample linearly increases with temperature rise. Also, by increasing the filler loading, the heat capacity decreases. Except for the sample with 5.4 vol% graphene loading.

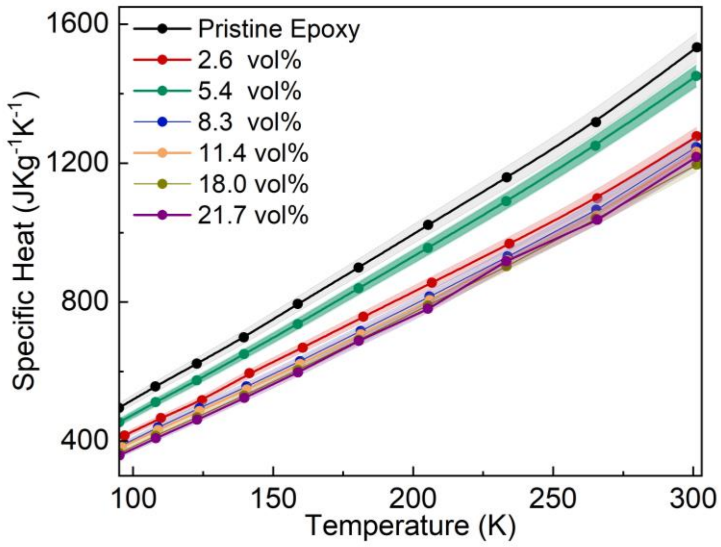

Supplementary Figure 4. Specific heat of graphene composites. Heat capacity of composites with different graphene loading as a function of temperature. The shaded area around the experimental data points displays the errors involved in the measurements.

5 Thermal conductivity measurements and uncertainty analysis

To measure the thermal conductivity, samples were cut into a typical dimension of 1×1×10 mm. A Quantum Design Physical Property Measurement System (PPMS) was employed to measure the thermal conductivity from 2 to 300 K with a steady-state 4-probe continuous mode. Data were accumulated continuously at a low heating rate of 0.3 Kmin^{-1}. Other measurement parameters such as heating power and period were adjusted by the software automatically.[2] When a square-wave heat pulse was applied, the temperature difference between the hot probe and cold probe was measured as a function of time, and a steady-state temperature difference was fitted using the equation below:

$$\Delta T = \Delta T_\infty \times (1 - \frac{\tau_1 \times \exp\left(-\frac{t}{\tau_1}\right) - \tau_2 \times \exp\left(-\frac{t}{\tau_2}\right)}{\tau_1 - \tau_2}) \qquad \text{(S5)}$$

Here, ΔT is the measured temperature difference, ΔT_∞ is the steady-state temperature difference, τ_1 and τ_2 are time constants and can be obtained from the fitting. When ΔT_∞ is calculated, the thermal conductance *K* is calculated as

$$K = P/\Delta T_\infty \tag{S6}$$

where *P* is the heat flowing through the sample. And *P* is calculated by subtracting the radiation loss from the Joule heating:

$$P = I^2 R - P_{rad} \tag{S7}$$

In this equation, *I* is the current flowing into the sample and *R* is the resistance of the heater. The radiation loss can be calculated as:

$$P_{rad} = \sigma_T \times (\frac{S}{2}) \times \varepsilon \times (T_{hot}^4 - T_{cold}^4) \tag{S8}$$

where σ_T = 5.67 × 10^{-8} $Wm^{-2}K^{-4}$ is the Stefan-Boltzmann constant, *S* is the surface area of the sample, ε is the emissivity, T_{hot} and T_{cold} are the temperature of the hot probe and cold probe. Then the shoe assembly's thermal conductance, K_{shoe}, is deduced from *K* to get the sample's thermal conductance, K_{sample}, as:

$$K_{sample} = K - K_{shoe} \tag{S9}$$

$$K_{shoe} = aT + bT^2 + cT^3 \tag{S10}$$

Here, *a*, *b*, and *c* are constants. The thermal conductivity of the sample is calculated as:

$$\kappa = K_{sample} \times l/(w \times t) \tag{S11}$$

where *l*, *w*, and *t* are the distance between the hot and cold probes, the width, and the thickness of the sample, respectively. The error of the thermal conductivity measurement originates from several factors such as errors in fitting of ΔT, and the errors in the heating power and in the estimation of the radiation loss due to the error of sample surface area and emissivity. Additionally, the errors in K_{shoe} and in the measurements of the size of the sample and the distance between hot and cold probes *d* should be considered. Therefore, the total error can be calculated as:

$$\sigma(\kappa) = \kappa \times \cdots \sqrt{(\frac{R_{\Delta T}}{\Delta T_{\infty}})^2 + (2\frac{IR\partial I}{P})^2 + (\frac{0.2 \times P_{rad}}{P})^2 + (\frac{0.1 \times T_{\infty} \times K_{shoe}}{P})^2 + (\frac{\Delta l}{l})^2 + (\frac{\Delta w}{w})^2 + (\frac{\Delta t}{t})^2}. \tag{S12}$$

In this equation, $R_{\Delta T}$ is the residual term from the fitting of the ΔT versus *t*.

6 Durability of composites at cryogenic temperatures

To verify the mechanical and thermal stability of the composite samples in the cryo-temperature ranges, we measured the thermal conductivity of the epoxy sample with 5.4 vol% filler loading three times from 2 K to 300 K. The surface morphology of the sample was inspected carefully after each temperature cycling via optical microscopy to track any possible development of cracks or other mechanical defects caused by thermal cycling. The results are presented in Supplementary Figure 5 (a). The inset shows the optical microscopy of the sample's surface after the third cycle. Supplementary Figure 5 (b) shows the same data in the temperature range between 2 K to 50 K. As seen, the thermal conductivity of the sample shows no significant changes due to the thermal cycling. In addition, in the inset of Supplementary Figure 5 (a), it is clear that the sample doesn't show any cracks after three cycles of measurements.

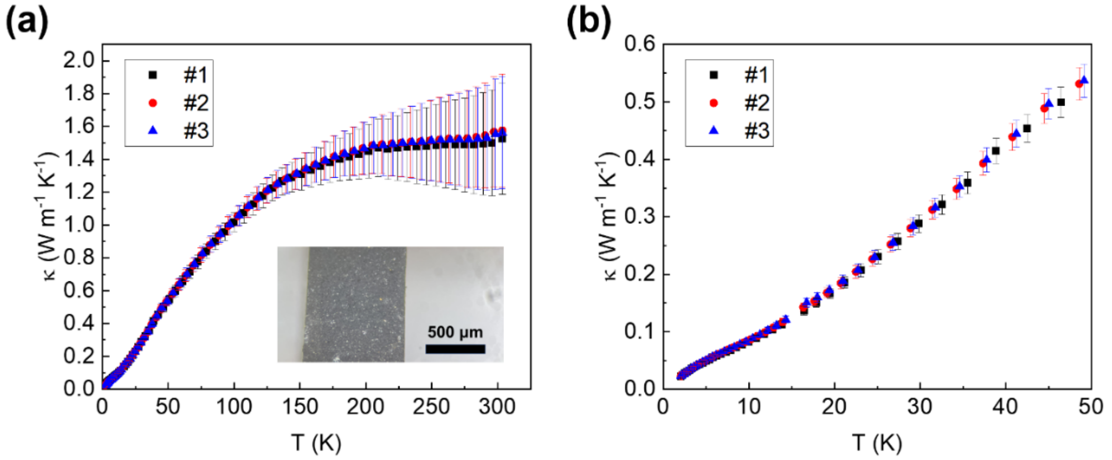

Supplementary Figure 5. Thermal stability of the graphene composites. (a) Thermal conductivity of the epoxy with 5.4 vol% graphene loading as a function of temperature cycling in the range of 2 K to RT. The inset shows an optical image of the sample after three measurements. No mechanical cracks were detected after several thermal cycling. (b) Thermal conductivity of the same sample shown in the cryogenic temperature range. As seen, the composite's thermal conductivity does not exhibit any changes after three times of thermal cycling.

7 Effective medium model for cryogenic heat conduction in low-loading composites

In equation (1) of the main text, L_{ii} are geometrical parameters and depend upon the aspect ratio, $p = t/L$, of graphene fillers with t and L being the thickness and lateral dimensions of fillers. For oblate inclusions such as nanoplatelets, where $p < 1$, these geometrical parameters, L_{ii}, are computed using the following equations,

$$L_{11} = L_{22} = \frac{p^2}{2(p^2 - 1)} + \frac{p}{2(1 - p^2)^{3/2}} \cos^{-1} p \tag{S13}$$

$$L_{33} = 1 - 2L_{11} \tag{S14}$$

In our models, we used the temperature-dependent in-plane and through-plane thermal conductivity data of graphite reported in Refs. [3,4]. These data are presented in Supplementary Figures 6 (a,b). It is clear that the thermal conductivity of graphite reaches very low values at low

temperatures. At 4 K, the in-plane thermal conductivity of graphite decreases to ~ 1 $Wm^{-1}K^{-1}$, while the through-plane thermal conductivity reaches 0.23 $Wm^{-1}K^{-1}$.

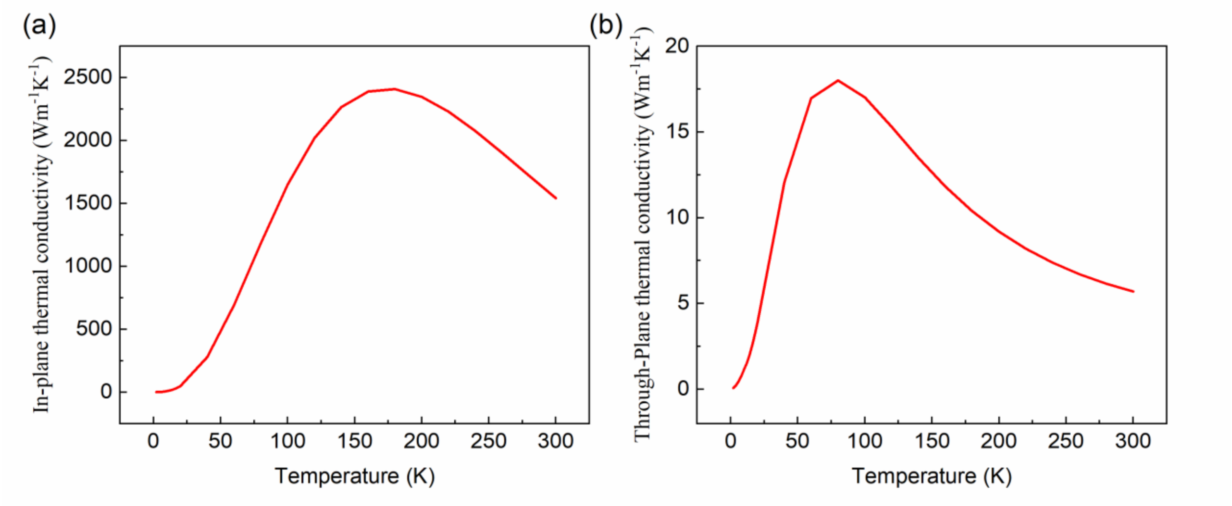

Supplementary Figure 6. Temperature-dependent thermal conductivity of pristine graphite. a) in-plane, and b) through-plane thermal conductivities of natural graphite as a function of temperature.

The explicit temperature dependence of the in-plane, k_{in}, and through-plane, k_{out}, thermal conductivities of graphite is given by the following equations. The equations are derived by fitting the data presented in Supplementary Figure 6 (a,b).

$$k_{in} = exp(0.00116[ln(T)]^6 - 0.02386[ln(T)]^5 + 0.17360[ln(T)]^4 - 0.66809[ln(T)]^3 + 1.67342[ln(T)]^2 - 0.19473[ln(T)]^1 - 1.65861). \quad (S15)$$

$$k_{out} = exp\ (0.0975538[ln(T)]^4 - 1.6222796[ln(T)]^3 + 9.1860571[ln(T)]^2 - 19.9450584[ln(T)]^1 + 14.4337754). \quad (S16)$$

The thermal conductivity of epoxy was taken to be temperature dependent from measurements. The following fittings to the experimental data can be used in the different temperature ranges.

For 2 K< T <14 K

$$k_{epoxy} = 1.5044 \times 10^{-6}T^5 - 7.2195 \times 10^{-5}T^4 + 1.3521 \times 10^{-3}T^3 - 1.2278 \times 10^{-2}T^2 + 5.5773 \times 10^{-2}T - 2.6347 \times 10^{-2} \quad \text{(S17)}$$

For 14 K < T <300 K

$$k_{epoxy} = 1.3105 \times 10^{-14}T^6 - 1.1288 \times 10^{-11}T^5 + 3.3133 \times 10^{-9}T^4 - 3.08119 \times 10^{-7}T^3 - 2.01387 \times 10^{-5}T^2 + 4.9861 \times 10^{-3}T + 1.13744 \times 10^{-2} \quad \text{(S18)}$$

Explicit temperature dependence of interface thermal resistance is provided below.

$$R_{interface} = \exp\left(0.0111[\ln(T)]^4 - 0.1147[\ln(T)]^3 + 0.3702[\ln(T)]^2 - 1.5114[\ln(T)]^1 - 8.7497\right) \quad \text{(S19)}$$

The interface thermal conductance and thermal resistance (inverse of conductance) are shown in Supplementary Figure 7.

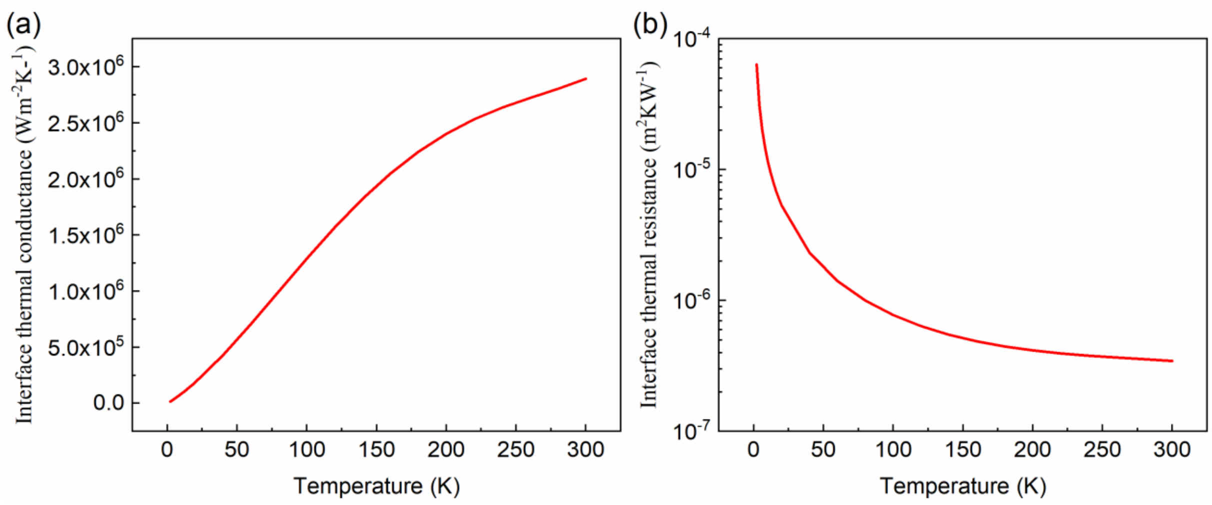

Supplementary Figure 7. Temperature-dependent interfacial thermal conductance. Interfacial a) thermal conductance, and b) thermal resistance between graphene and pure epoxy as a function of temperature.

8 Effective medium model for cryogenic heat conduction in high-loading composites

In equation (5) of the main text, S_{11} and S_{33} are the shape parameters related to the aspect ratio of graphitic nanosheets, given by the following equations.

$$S_{11} = S_{22} = \frac{p}{2(1-p^2)^{3/2}}\left[\cos^{-1} p - p(1-p^2)^{1/2}\right], \qquad p < 1 \tag{S20}$$

$$S_{33} = 1 - 2S_{11} \tag{S21}$$

In the above equations, p is the aspect ratio of the fillers. The effective in-plane and through-plane thermal conductivities, k_{11} and k_{33} (in Equation (5) in the main text) are computed using

$$k_{11} = k_0[1 + \frac{(1-c_{int})(k_{in}-k_0)}{c_{int}S_{11}(k_{in}-k_0)+k_0}] \tag{S22}$$

$$k_{33} = k_0[1 + \frac{(1-c_{int})(k_{out}-k_0)}{c_{int}S_{33}(k_{out}-k_0)+k_0}] \tag{S23}$$

In the above equations, k_0 is the thermal conductivity of an interlayer surrounding graphene sheets. This interlayer represents the interface thermal resistance surrounding the graphene particles and is used to model the combined effect of graphene-epoxy and graphene-graphene contact resistance. c_{int} represents the volume concentration of interlayer with respect to the entire coated graphene filler. The values of different parameters used in the percolation-based effective medium model are described below. Note that we used temperature-dependent properties wherever it was needed. Supplementary Table 1 shows the values of different parameters used in the above calculations.

Supplementary Table 1: Thermal conductivity calculations parameters

Material Parameters	Values
Average graphene lateral length, l,	15 μm
Average graphene thickness	15 nm
Aspect ratio of the graphene filler	0.001
Thermal conductivity of epoxy	Temperature dependent
Thermal conductivity of graphene filler, k_1 and k_3 (W/mK)	Temperature dependent
Thermal conductivity of interlayer with Kapitza resistance	Temperature dependent
Thermal conductivity of the interlayer with a firmly developed graphene-graphene contact state,	Temperature dependent

The in-plane, k_{in}, and through-plane thermal conductivities, k_{out}, of graphene were taken to be temperature dependent as shown in Supplementary Figure 6 (a,b). The combined interfacial resistance of graphene/epoxy and graphene/graphene contact is modeled as an interlayer in the above theory with an effective thermal conductivity of k_0 which is taken to be a weighted sum of the thermal conductivity of graphene/epoxy and graphene/graphene contact. The thickness of this interlayer was nominally taken to be 1.0 nm. Thermal conductivity of the interlayer based on graphene-epoxy contact was computed from the thickness and interfacial resistance of graphene/epoxy contact which is taken to the same as given by Equation (S19). The thermal conductivity of interlayer based on graphene/graphene contact was computed from the interfacial resistance at graphene-graphene contact which was taken to be lower than graphene-epoxy interfacial thermal resistance by a factor ranging from 4.5 for 11.4 vol% compositions to 6.0 for 21.7 vol% compositions.

Supplementary References

1. Lashley, J. C. *et al.* Critical examination of heat capacity measurements made on a Quantum Design physical property measurement system. *Cryogenics* **43**, 369–378 (2003).
2. Maldonado, O. Pulse method for simultaneous measurement of electric thermopower and heat conductivity at low temperatures. *Cryogenics* **32**, 908–912 (1992).
3. Nakamura, S., Miyafuji, D., Fujii, T., Matsui, T. & Fukuyama, H. Low temperature transport properties of pyrolytic graphite sheet. *Cryogenics* **86**, 118–122 (2017).
4. Ho, C. Y., Powell, R. W. & Liley, P. E. Thermal Conductivity of the Elements. *J. Phys. Chem. Ref. Data* **1**, 279–421 (1972).
